# Epidemiology and outcomes of 655 diabetic foot patients in a Brazilian university hospital

**DOI:** 10.1186/1758-5996-7-S1-A20

**Published:** 2015-11-11

**Authors:** Ligia de Loiola Cisneros, Rafael Henrique Rodrigues Costa, Tulio Pinho Navarro

**Affiliations:** 1 Universidade Federal de Minas Gerais, Belo Horizonte, Brazil

## Background

Diabetic foot infections are a feared diabetic complication once it is associated to high amputation rates. The vascular surgeon plays a special role assessing and treating macrovascular impairments in order to avoid major amputations and death.

## Objective

Assessment of the epidemiological data and outcomes –rates of mortality, hospital readmissions and limb salvage-of patients with diabetic foot infections treated in a tertiary university hospital in Brazil.

## Materials and methods

From January/2007 to December/2012, 655 patients with diabetic foot infections or ulcers were admitted at the vascular surgery unit. Retrospective medical records were reviewed and analyzed. The predictors for lower limb amputation and death were determined using the conditional logistic regression model analysis.

## Results

Sixty seven percent (442) were males; the age ranged from 21 to 102 years (median 63 years). Arterial ischemia was present in 28% of the patients. Among these diabetic patients 73% had hypertension and 30% were active smokers. The in-hospital mortality rate was 12%, and there was no statistically difference between patients with ischemic and non-ischemic lesions (P=0.16). Of the 576 patients alive, 61% were not readmitted, 21% were readmitted once and 18% were readmitted twice or more times. The minor amputation rate was 48% while major amputations were performed in 21% of the subjects (28% below the knee amputation and 72% above the knee amputation). The major amputation free survival rate was 72%. After discharge 47% of the patients required special home-care for dressings and for parenteral drug administration. Independent risk factor for amputation were age (OR: 1.02; 95%CI: 1.001-1.035; P=0.041) and arterial ischemia (OR 2.20; 95%CI 1.46-3.31; P<0.0001). Independent risk factors for death were age (OR 1.06; 95% CI 1.03 – 1.08; P<0.01) and major amputations (OR 2.38; 95% CI 1.41 – 3.99; P=0.01).

## Conclusion

Diabetic foot is a severe condition with high mortality and amputation rates. Conditions associated with limb loss were age and ischemia. The independent risk factors for death were age and major amputation.

